# Ethanolic Leaf Extract of *Annona muricata* Pauses *Plasmodium knowlesi* Schizogony and Reduces Binding of Infected Red Blood Cells to Endothelial Cells

**DOI:** 10.3390/tropicalmed11070184

**Published:** 2026-07-06

**Authors:** Yi-Jun Lim, Gordon Xue-Zhen Chong, Joo-Yie Chin, Muhammed-Nur-Iman Mohammed-Syafiei, Muhammad-Nasreen Suhaimi, Siti-Nursyazziana Nordin, Shin-Yee Fung, Hazel Anne Tabo, Polrat Wilairatana, Tadesse Hailu, Veeranoot Nissapatorn, Wenn-Chyau Lee

**Affiliations:** 1 Department of Parasitology, Faculty of Medicine, Universiti Malaya, Kuala Lumpur 50603, Malaysia; limyijun9@gmail.com (Y.-J.L.); gordonchong80@gmail.com (G.X.-Z.C.); jooyie10@um.edu.my (J.-Y.C.); iman176999@gmail.com (M.-N.-I.M.-S.); muhammadnasreen15@gmail.com (M.-N.S.); ziananordin03@gmail.com (S.-N.N.); 2 Department of Molecular Medicine, Faculty of Medicine, Universiti Malaya, Kuala Lumpur 50603, Malaysia; syfung@um.edu.my; 3 Biological Sciences Department, College of Science and Computer Studies, De La Salle University-Dasmariñas City, Cavite 4115, Philippines; hltabo@dlsud.edu.ph; 4 Department of Clinical Tropical Medicine, Faculty of Tropical Medicine, Mahidol University, Bangkok 10140, Thailand; polrat.wil@mahidol.ac.th; 5 Department of Medical Parasitology and Entomology, College of Medicine and Health Sciences, Bahir Dar University, Bahir Dar P.O. Box 79, Ethiopia; tadessehailu89@gmail.com; 6 Futuristic Science Research Center, School of Science, World Union for Herbal Drug Discovery (WUHeDD), Research Excellence Center for Innovation and Health Products (RECIHP), Walailak University, 222 Thaiburi, Thasala District, Nakhon Si Thammarat 80160, Thailand; 7 A*STAR Infectious Diseases Labs (A*IDL), Agency for Science, Technology and Research (A*STAR), Singapore 138648, Singapore

**Keywords:** malaria, *Plasmodium knowlesi*, soursop, endothelial binding, parasitostatic, vaso-protective

## Abstract

*Plasmodium knowlesi* infections can progress rapidly to life-threatening complications, such as acute respiratory distress syndrome (ARDS) and acute kidney injury (AKI), which are driven by the ability of the zoonotic parasite to rapidly replicate towards high parasitemia and the cytoadherence properties of the infected erythrocytes (IRBCs). This study evaluated the anti-parasitic and vasoprotective potential of *Annona muricata* (soursop) leaf ethanol extract against *P. knowlesi*. In vitro assays using the *P. knowlesi* A1-H.1 reference strain revealed significant blood stage schizogony inhibition (IC_50_: 9.65 µg/mL), specifically targeting the trophozoites. The anti-parasitic activity was concentrated in the <30 kDa subfraction of the extract. Washout assays confirmed that the effect was parasitostatic rather than parasiticidal, where the malaria parasites underwent developmental arrest but remained morphologically normal and resumed growth post-removal. Furthermore, priming human endothelial cell lines with the extract significantly reduced IRBC–endothelial binding. These results demonstrate that *A. muricata* extract exerts a dual-action effect by arresting *P. knowlesi* asexual replication and inhibiting IRBC–endothelial cytoadherence. While clinical translation would require exhaustive standardization and chemotypic profiling due to the natural variability of plant compositions, these findings provide a foundational academic framework for the potential of *A. muricata* leaf extract in mitigating severe knowlesi malaria complications.

## 1. Introduction

*Plasmodium knowlesi*, a zoonotic parasite naturally found in long-tailed macaques, has emerged as the primary cause of human malaria in Southeast Asia [1], particularly in the forested areas that harbor simio-anthropophagic anopheline vectors [2,3,4,5]. Characterized by a rapid 24 h asexual cycle, *P. knowlesi* can lead to hyperparasitemia and life-threatening complications, most notably acute respiratory distress syndrome (ARDS) and acute kidney injury (AKI) [6,7,8,9,10]. These severe manifestations are increasingly linked to the sequestration of infected red blood cells (IRBCs) within the microvasculature, driven by cytoadherence to endothelial cells in target organs [8,10]. For at-risk populations in remote forested regions, the rapid progression from symptom onset to multi-organ failure, as well as the difficulty for these patients to get access to the well-resourced healthcare centers in towns, presents a critical clinical challenge [11].

While artemisinin-based combination therapies (ACTs) remain the gold standard [12,13], there is an urgent need for adjunctive therapies that can specifically target the vascular pathogenesis of the disease. *Annona muricata* (soursop) is a promising candidate; its leaf extracts have demonstrated potent anti-plasmodial activity against *Plasmodium* in traditional and laboratory settings [14,15,16]. In addition to its anti-parasitic effects, the extract exhibits anti-inflammatory and vasoprotective properties that could mitigate organ damage [15,17,18]. Nevertheless, the effect of *A. muricata* leaf extracts on the unique virulence factors of *P. knowlesi* remains unexplored. This study evaluated the efficacy of an ethanolic leaf extract of *A. muricata* against *P. knowlesi* growth and its potential to inhibit IRBC binding to human pulmonary microvascular (HPMEC) and renal glomerular (HRGEC) endothelial cells.

## 2. Materials and Methods

### 2.1. Materials Used and General Experimental Conditions

Detailed information on materials used in the experiments is available in Supplementary Materials Table S1. All experimental procedures were conducted in strict accordance with the guidelines of the Universiti Malaya Institutional Biosafety and Biosafety Committee (IBBC; ref: UMIBBC/PA/R/FOM/PARA-025/2022) and the University of Malaya Medical Centre (UMMC) Medical Research Ethics Committee (MREC ID: 2024312-13526). Unless otherwise specified, all experiments were performed using eight biological replicates.

### 2.2. In Vitro Cultures of Malaria Parasites and Human Endothelial Cell Lines

Reference strains of *P. knowlesi* (A1-H.1) were maintained in RPMI 1640 medium supplemented with AlbuMAX II and 10% (*v*/*v*) heat-inactivated human serum (complete parasite culture medium) [8,19,20]. Cultures were incubated under a tri-gas atmosphere (5% CO_2_, 5% O_2_, and 90% N_2_) at 37 °C. Before experiments, parasites were stage-synchronized using magnetic-activated cell sorting (MACS) LD columns (Miltenyi Biotec, Bergisch Gladbach, Germany), where the late-stage parasites with high accumulation of hemozoin were retained by the magnetic field of the sorting system [21]. For human endothelial cell cultivation, culture flasks were pre-coated with 0.5% gelatin solution for 6 h at 37 °C. Subsequently, the human pulmonary microvascular (HPMEC) and human renal glomerular (HRGEC) endothelial cell lines were thawed and inoculated into the prepared flasks under in vitro cultivation conditions supplied with 5% CO_2_ [10].

### 2.3. Drug Plate Preparation and Parasite Susceptibility Evaluation

The *A. muricata* leaves were subjected to ethanolic extraction using established extraction protocols described elsewhere [22,23]. Briefly, fresh, mature, and healthy *A. muricata* leaves were collected from Laguna, the Philippines. Species identification was confirmed by Dr. Sandra Yap, a botanist from the Far Eastern University Herbarium (FEUH), the Philippines, as *A. muricata* L., family Annonaceae, with specimen accession number 2025-104A. The collected leaves were cleaned with distilled water to remove debris prior to air-drying and pulverization. The powdered leaves (200 g) were soaked in 800 mL of 95% ethanol for 5 days at 25 ± 2 °C with occasional stirring. The crude extract was formed following filtration and rotary evaporation.

Drug assays were performed in flat-bottom, 96-well microplates with a total culture volume of 200 µL per well [24,25,26]. Two types of drug plates were prepared: a dihydroartemisinin (DHA)-seeded plate and an *A. muricata* leaf extract-seeded plate. To prepare the DHA plates, the drug was dissolved in absolute, non-denatured ethanol to achieve final test concentrations ranging from 0 to 703.4 nM. The solution was added to the wells, air-dried under sterile conditions, and stored at −20 °C until use [27,28]. Similarly, the *A. muricata* leaf extract was prepared in absolute ethanol and added to separate plates to achieve final working concentrations of 0 to 640 µg/mL, based on the previous study against *P. falciparum* [14]. For untreated control wells, an equal volume of absolute ethanol was added and dried under identical conditions.

The schizont maturation inhibition assay was conducted with stringently stage-synchronized *P. knowlesi*, adapted from protocols previously described [24,28,29]. Briefly, the parasite (IRBCs with at least 80% of the parasite population in the fine, early (ring) stage, parasitemia adjusted to 1%, prepared as a 2% hematocrit suspension in the complete parasite culture medium) was subjected to the exposure of DHA and *A. muricata* leaf extract and cultured in the tri-gas atmosphere until the parasites in the drug-free control wells reached the mature schizont stage (consisting of at least eight merozoites per schizont under the Giemsa-stained blood smear). Thick and thin smears were made from each tested well, stained with 5% Giemsa solution, and examined with a light microscope under immersion oil magnification. The percentage of parasite population that reached the mature schizont stage was determined by recruiting 200 parasites.

We also compared the susceptibility of *P. knowlesi* early and late erythrocytic stages to the leaf extract. Based on the inhibitory concentration of the leaf extract obtained from the schizont maturation inhibition assay, a separate experiment was conducted, where the ring stage parasites were incubated with *A. muricata* leaf extract (320 µg/mL) for two hours under in vitro cultivation conditions. Subsequently, the leaf extract was removed via centrifugation and removal of supernatant, followed by suspending the pelleted cells with fresh *Plasmodium* culture medium and culturing them under the in vitro cultivation conditions until the parasites in the drug-free controls reached the mature schizont stage. The experiment was repeated with late-stage parasites (mid- to late-stage trophozoite) to investigate the susceptibility of the parasite early and late stages to the *A. muricata* leaf extract [28].

### 2.4. Molecular Weight-Based Fractionation of A. muricata Leaf Extract

The *A. muricata* leaf extract starting solution (320 µg/mL) was separated based on the molecular weight of solutes within it, using a Vivaspin20 twin PES membrane concentrator (30,000 MWCO) (Sartorius, Göttingen, Germany) centrifuged at 3000× *g* for 20 min [30]. This process yielded a filtrate (<30 kDa) and a retentate (≥30 kDa) fraction. To assess their relative contribution to the crude extract’s potency, both fractions were tested in the schizont maturation inhibition assay at volumes equivalent to their respective yields from the initial 320 µg/mL extract. In a separate assay tracking the growth kinetics post-exposure, parasites were treated with an untreated control, a solvent control (200 µL absolute ethanol per well, dried completely under the same conditions as the drug plate preparation), 703.4 nM DHA, and the volumetric equivalents of the <30 kDa and ≥30 kDa fractions. Following incubation, the parasite suspension from each well was collected, centrifuged, and replenished with fresh culture medium. The cultures were maintained for an additional 72 h at 37 °C under the tri-gas atmosphere. Parasitemia was monitored every 24 h using the Giemsa-stained thin blood smears to observe the long-term parasite population recovery.

### 2.5. Effect of A. muricata Leaf Extract on P. knowlesi-Infected Erythrocyte (IRBC)–Endothelial Cytoadherence

The *P. knowlesi*–IRBCs have been demonstrated to cytoadhere to certain types of human endothelial cells (particularly the pulmonary microvascular and renal glomerular endothelial cells), which aligns with the commonly reported severe complications of knowlesi malaria [8,10]. To test the effect of *A. muricata* leaf extract on the cytoadherence dynamics of *P. knowlesi*–IRBCs with endothelial cells, each of the chamber slide-seeded endothelial cell lines (HPMECs and HRGECs) was divided into two groups. One group was co-incubated with the leaf extract (320 µg/mL) for 24 h under in vitro cultivation conditions, and the other group served as the “unprimed control”. Subsequently, the cell lines were co-incubated with *P. knowlesi* culture suspension (at least 70% of the parasite population being the late-stage parasites) for two hours prior to gentle washing steps to remove unbound cells from the surface of the endothelial cells. After that, the cells were fixed with ice-cold absolute methanol for ten minutes, followed by staining with Giemsa, and they were examined via light microscopy with immersion oil magnification. The IRBC–endothelial binding was calculated by determining the number of IRBCs attached to endothelial cells per 100 fields (equivalent to the coverage of approximately 8000 cells) [8].

### 2.6. Statistical Analyses

GraphPad Prism 11.0 was used for data analyses. The normality of the data was determined with the Shapiro–Wilk test. For unpaired comparison of two sets of parametric data, Welch’s *t*-test was used. For the comparison of multiple matched, non-parametric datasets, the Friedman test with Dunn’s multiple comparison analysis was used. For the comparison of multiple independent, non-parametric data, the Kruskal–Wallis test with Dunn’s multiple comparison analysis was used. To determine the IC_50_ of the compound, non-linear X-Y analysis was used. For the comparison of two categorical independent factors (duration and types of compound exposed) on a continuous dependent variable (parasitemia), two-way ANOVA with Tukey’s test was applied.

## 3. Results

### 3.1. Ethanol Extract of A. muricata Leaf Significantly Suppressed Erythrocytic Schizogony of P. knowlesi

**Figure 1:**
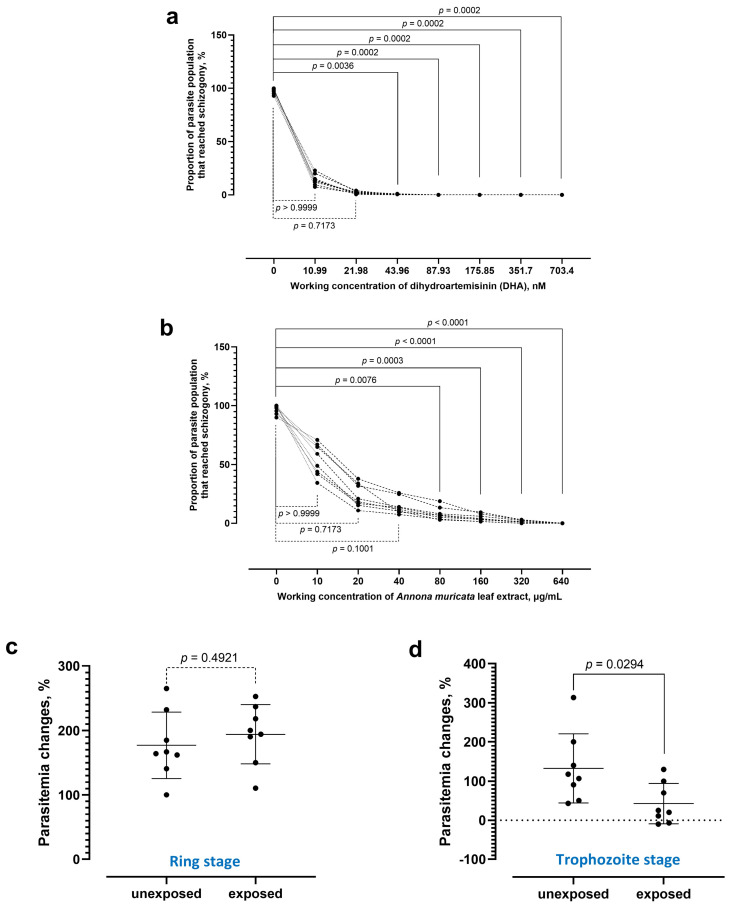
Susceptibility of *P. knowlesi* via in vitro assays. (**a**) Schizont inhibition assay with dihydroartemisinin (DHA). Based on the Friedman test with Dunn’s multiple comparison test, the schizogony of *P. knowlesi* was significantly halted at concentration points of 43.96 nM DHA and above (*p* < 0.0001; Q = 54.06, *n* = 8, df = 7), as compared to the drug-free control. (**b**) Schizont inhibition assay with *A. muricata* leaf extract. Based on the Friedman test with Dunn’s multiple comparison test, the schizogony of *P. knowlesi* was significantly halted at concentration points of 80 µg/mL and above (*p* < 0.0001; Q = 55.87, *n* = 8, df = 7), as compared to the drug-free control. Additionally, there was no significant difference in the proportion of parasite population that reached schizogony between experiment groups 320 µg/mL and 640 µg/mL *A. muricata* leaf extract (*p* > 0.9999). (**c**) Welch’s *t*-test revealed that the two-hour exposure of the *P. knowlesi* ring stage to 320 µg/mL *A. muricata* leaf extract did not significantly alter the parasite population growth, as compared to the unexposed control (Welch-corrected t = 0.7057, df = 13.82). Error bars represent mean and SD. (**d**) Welch’s *t*-test revealed that the two-hour exposure of the *P. knowlesi* trophozoite stage to 320 µg/mL *A. muricata* leaf extract significantly reduced the parasite population growth, as compared to the unexposed control (Welch-corrected t = 2.492, df = 11.28). Error bars represent mean and SD.

From the schizont maturation inhibition assay, the schizogony of *P. knowlesi* was significantly hampered by DHA (Figure 1a), with an IC_50_ of 1.70 nM (95% CI 1.40–2.02 nM). The asexual replication of *P. knowlesi* was also hampered by *A. muricata* leaf extract (Figure 1b), where the proportion of the parasite population that reached schizogony was significantly reduced at working concentrations of 80 µg/mL and above, as compared to the drug-free control. The IC_50_ of the *A. muricata* leaf extract against *P. knowlesi* was 9.65 µg/mL (95% CI 7.86–11.78 µg/mL). The leaf extract at working concentrations of 320 µg/mL and 640 µg/mL demonstrated a similar inhibitory effect against the parasites. Subsequent testing with 320 µg/mL of *A. muricata* leaf extract revealed that the leaf extract was ineffective against the ring stages of the parasites (Figure 1c). Instead, the leaf extract was potent against the trophozoite stages of *P. knowlesi* (Figure 1d). Succinctly, the ethanol extract of *A. muricata* leaf was potent against the asexual replication of *P. knowlesi* blood stages.

### 3.2. Schizogony-Halting Effect Attributed to Extract Compounds Smaller than 30 kDa

**Figure 2:**
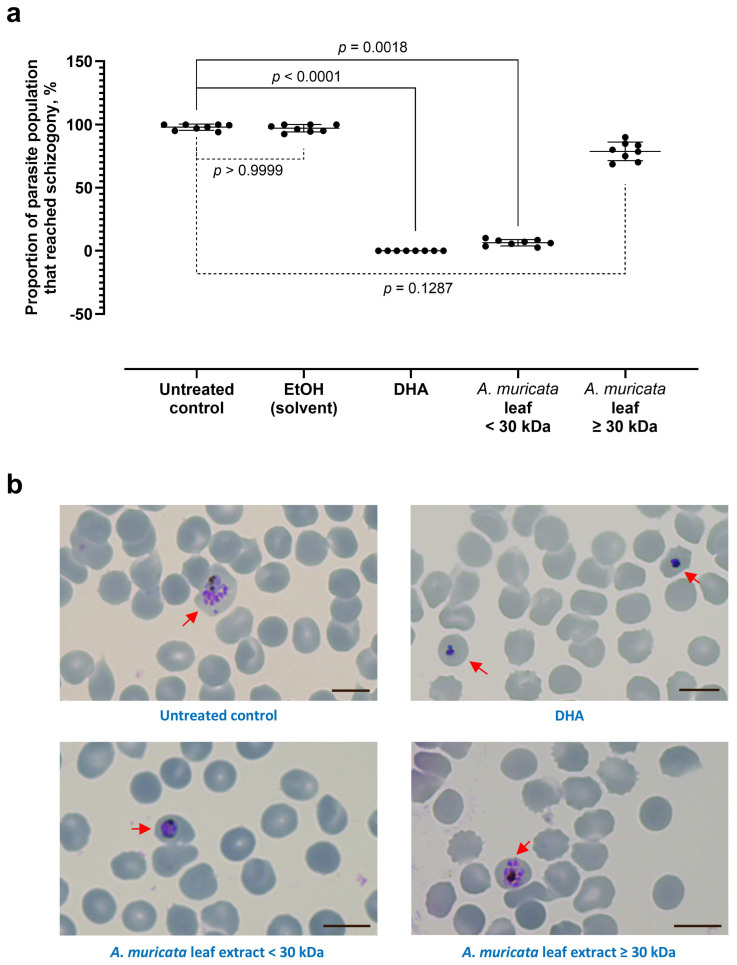
Deciphering subfractions of the *A. muricata* leaf extract with potent hampering effect on *P. knowlesi* growth. (**a**) A Kruskal-Wallis test with Dunn’s multiple comparison test revealed that the subfraction of <30 kDa demonstrated a significant blocking effect on *P. knowlesi* schizogony, as compared to the untreated control (*p* < 0.0001; *H* = 35.75, df = 4). Error bars represent means and SD. (**b**) Morphology of *P. knowlesi* upon the end of the schizont maturation inhibition assay under different experimental settings, visualized with 5% Giemsa-stained thin blood smears and examined with light microscopy under immersion oil magnification. The IRBC is indicated by red arrow. Scale bar = 10 µm.

The *A. muricata* leaf extract was further fractionated based on the molecular weight of the compounds within. We found that the anti-parasitic effect of the *A. muricata* leaf extract was attributed to the fraction of <30 kDa (Figure 2a). The *A. muricata* leaf extract of ≥30 kDa caused insignificant changes to the proportion of parasite population that reached schizogony, as compared with the untreated control. Notably, parasites exposed to the active fraction of *A. muricata* displayed a distinct morphology compared to DHA-treated parasites. While DHA-treated parasites were pyknotic (indicating cell death), those exposed to the <30 kDa extract showed arrested development without chromatin pyknosis (Figure 2b). This suggests that the extract induces a reversible growth inhibition rather than immediate parasiticidal action.

### 3.3. A. muricata Leaf Extract Was Parasitostatic Instead of Parasitocidal

**Figure 3:**
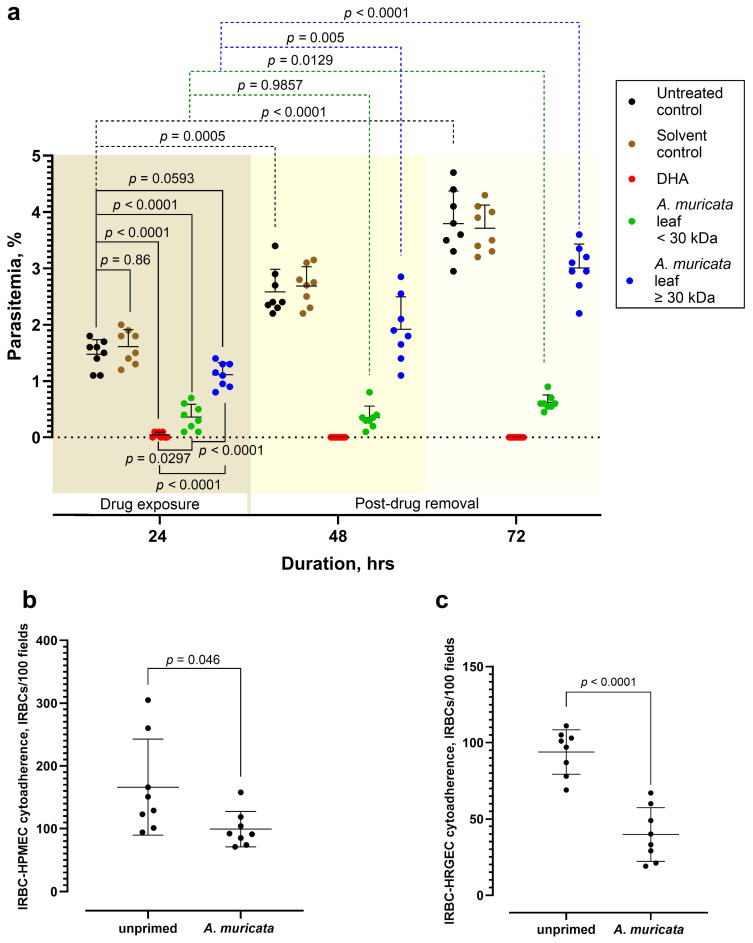
Further characterization of the effect of *A. muricata* leaf extract on pathobiology of *P. knowlesi* infection. (**a**) The monitoring of parasitemia changes following 24 h of incubation with drugs (DHA, *A. muricata* leaf extract <30 kDa subfraction, *A. muricata* leaf extract ≥30 kDa subfraction, solvent control, and untreated control), and an additional 48 h after removal of the tested components, as indicated by the background colors of the graph. Based on the two-way ANOVA with Tukey’s test, significance was found with regard to the duration monitored (F_1.72,60.28_ = 211.4, *p* < 0.0001) and the types of compounds exposed to the parasites (F_4,35_ = 229.6, *p* < 0.0001). There was a significant interaction between the duration monitored and the types of compounds exposed to the parasites (F_6.89,60.28_ = 30.65, *p* < 0.0001). Comparisons between the data collected right after drug exposure and the data collected post-drug removal were indicated with dotted lines. (**b**) Priming of HPMECs with the *A. muricata* leaf extract significantly reduced the binding of *P. knowlesi*–IRBCs to the endothelial cells (Welch-corrected t = 2.317, df = 8.884). (**c**) Priming of HRGECs with the *A. muricata* leaf extract significantly reduced the binding of *P. knowlesi*-IRBCs to the endothelial cells (Welch-corrected t = 6.695, df = 13.50). Error bars represent means and SD.

To validate the parasitostatic nature of the extract, we exposed the parasites to both fractions of the *A. muricata* leaf extract, along with DHA and drug-free controls for 24 h, which demonstrated a significant growth inhibitory effect against the parasites by DHA and the *A. muricata* leaf extract fraction of <30 kDa (Figure 3a). Subsequently, the tested materials were removed from the system, replenished with fresh parasite culture medium, and monitored closely. After removing the *A. muricata* leaf extract fraction of ≥30 kDa, the parasites continued to replicate, causing a significant increment in parasitemia 24 h post-removal of the tested material (i.e., the 48th hour of the experiment). For the group exposed to the *A. muricata* leaf extract fraction of <30 kDa, the parasites were “revived” at a slower pace, with a significant increment in parasitemia recorded 48 h post-removal of the *A. muricata* leaf extract fraction of <30 kDa from the system. Of note, the parasites appeared morphologically normal. The experimental group exposed to DHA showed no parasite growth after the drug removal from the system, indicating successful killing of the parasites by DHA. In short, the active ingredients within the *A. muricata* leaf extract exerted growth arrest on *P. knowlesi* instead of killing the parasites.

### 3.4. A. muricata Leaf Extract Reduced IRBC–Endothelial Binding

The lung-derived HPMEC primed with the *A. muricata* leaf extract prior to exposure to *P. knowlesi* demonstrated significantly lower IRBC–endothelial binding, as compared to the unprimed HPMEC (Figure 3b). Similarly, prior exposure to the *A. muricata* leaf extract caused a significant reduction in *P. knowlesi*–IRBC cytoadhesion to the kidney-derived HRGEC (Figure 3c).

## 4. Discussion

Our findings of the *P. knowlesi* schizogony-blocking effect by the *A. muricata* leaf ethanol extract agreed well with the earlier studies with *P. falciparum* [14]. We further showed that the schizogony-hampering effect by the *A. muricata* leaf extract was potent against the mature forms of the parasite erythrocytic stages (i.e., trophozoite) but ineffective against the younger forms (i.e., ring). Based on studies conducted with *P. falciparum*, several parasite-derived proteins play crucial roles during the transformation of trophozoite to schizont. These include the ribosomal P2 protein [31], SUN domain proteins [32], merozoite organizing protein [33], cell cycle regulatory proteins [34,35], and farnesyltransferase [36,37]. The *A. muricata* leaf-derived potent players may block the maturation of trophozoite to schizont via interactions with these parasite-derived proteins. Notably, the potent anti-schizogony compounds in the *A. muricata* leaf extract were of a molecular weight smaller than 30 kDa. The filtering of ethanol leaf extract with a PES membrane column of a 30 kDa cutoff value yielded a “cleaner” extract subfraction without large proteins and enzymes, complex polysaccharides, and large condensed tannins. The *A. muricata* leaf extract subfraction of <30 kDa contains smaller components such as alkaloids, flavonoids, phenolic compounds, and small tannin oligomers [38,39,40,41]. These metabolites are known for their anti-inflammatory and enzyme-inhibitory properties, which likely mediate the observed growth arrest [17,39,42]. The molecular weight-based fractionation using a 30 kDa cutoff membrane served as an initial chemical characterization barrier to differentiate the biological impact of macro-structural components from low-molecular-weight secondary metabolites. Many of the herbal extract-derived metabolites with therapeutic potential (such as acetogenins, flavonoids, and alkaloids) possess molecular weights well below the 30 kDa threshold [13,14]. However, there are larger molecular weight components (such as tannins, polyphenolic polymers, and certain polysaccharides) that contribute to antimicrobial and oxidation-modulating effects [14,37,38]. The fundamental purpose of utilizing a 30,000 MWCO membrane filter was not to partition individual micro-molecules but rather to isolate the active low-molecular-weight fractions from these large, complex botanical macro-aggregates, condensed tannins, or proteins (>30 kDa) that frequently cause non-specific mechanical interference in the in vitro microplate bioassays. This step confirmed that the observed anti-schizogony effect is driven explicitly by the small-molecule phytochemical matrix. Further characterization with techniques such as ultra-high-performance liquid chromatography (UHPLC) and direct chemical comparisons is warranted to further decipher the potent components against *Plasmodium* schizogony, followed by subsequent specific mechanism-deciphering investigations.

Importantly, the *A. muricata* leaf extract did not kill the malaria parasites. Instead, it induced a state of developmental arrest in the parasites, preventing them from completing the asexual replication that releases numerous merozoites, each of which is capable of invading uninfected erythrocytes [43]. This parasitostatic effect may serve as a critical “buy time” strategy for knowlesi malaria patients from remote, hard-to-reach forested areas, allowing them a window to reach urban healthcare facilities for the gold standard management of malaria [1]. Hence, the leaf extract of this widely available plant in the tropics may lower the risk of developing severe malaria complications among malaria patients. In addition to the parasitostatic effect, the *A. muricata* leaf extract also demonstrated a malaria-specific vasoprotective effect by reducing the *P. knowlesi*–IRBC cytoadherence to the endothelial cells. The lowering of IRBC–endothelial cytoadherence reduces endothelial activation, hence vascular injury, which is the hallmark of severe malaria pathogenesis [43]. In this study, cytotoxicity evaluation of the *A. muricata* leaf extract on the endothelial cell lines was not performed, as earlier studies have demonstrated the low toxicity of this extract on different human cell lines, including the endothelial cell lines [44,45,46]. Nevertheless, a formal, parallel cell line cytotoxicity assay would be a valuable addition to evaluate the extract’s therapeutic potential. Of note, brain-derived endothelial cell lines were not used in our study, as cerebral malaria or neurological complications are rare among the knowlesi malaria patients [47,48]. Our findings suggest that the application of the *A. muricata* leaf extract may reduce the risk of knowlesi malaria patients from developing severe complications such as ARDS and AKI. Although the *A. muricata* leaf extract has been shown to possess good tolerability and safety [49], further experimentation is needed to translate this into clinical application. In addition, the potential of this extract to reverse artemisinin resistance by the artemisinin-resistant *P. falciparum* isolates deserves to be investigated in the future.

## 5. Conclusions

The *A. muricata* leaf ethanol extract demonstrated a parasitostatic effect against *P. knowlesi* and significantly reduced the cytoadherence of IRBCs to lung- and kidney-derived human endothelial cells. These dual anti-parasitic and vasoprotective properties suggest that the extract has the potential to serve as an adjunctive therapy to mitigate the risk of severe complications, such as ARDS and AKI, in knowlesi malaria patients.

## Data Availability

The data presented in this study are available in this article and its Supplementary Materials.

## Supplementary Materials

The following supporting information can be downloaded at: https://www.mdpi.com/article/10.3390/tropicalmed11070184/s1, Table S1: Information on reagents and biological materials used in this study.

## Abbreviations

The following abbreviations are used in this manuscript:

DHA	Dihydroartemisinin
HPMECs	Human Pulmonary Microvascular Endothelial Cells
HRGECs	Human Renal Glomerular Endothelial Cells
IRBCs	Infected Red Blood Cells
