# Supplementary material for: Ethanolic Leaf Extract of Annona muricata Pauses Plasmodium knowlesi Schizogony and Reduces Binding of Infected Red Blood Cells to Endothelial Cells
Source: Trop Med Infect Dis. 2026 Jul 6;11(7):184. doi: 10.3390/tropicalmed11070184 (PMC13417136; doi:10.3390/tropicalmed11070184)
Supplement: Supplementary file 1 [file tropicalmed-11-00184-s001.zip › tropicalmed-4347458-supplementary.pdf]

Supplementary Material

Table S1. Information on reagents and biological materials used in this study.

Reagent/Resource	Reference or source	Identifier/ Catalog Number / Notes
**Biological materials**		
A1-H.1 (*Plasmodium knowlesi*)	Laboratory-adapted parasite strain; maintained in Universiti Malaya.	Used for all experiments
Human Pulmonary Microvascular Endothelial Cell (HPMEC)	ScienCell™ Research Laboratories; maintained in Universiti Malaya.	Cat#3000; used for all cytoadherence assay
Human Renal Glomerular Endothelial Cell (HRGEC)	ScienCell™ Research Laboratories; maintained in Universiti Malaya.	Cat#4000; used for all cytoadherence assay
**Chemicals, Enzymes and other reagents**		
1× phosphate buffer saline (PBS)	Gibco™	Cat#20012-027
99.8% ethyl alcohol (non-denatured)	Systerm	Cat#ET105-50
AlbuMAX II™	Gibco™ ThermoFisher Scientific	Cat#11021-037
Annona muricata extract	Prepared in WalailakUniversity.	-
D-glucose	Sigma-Aldrich®	Cat#G7520-1KG
Dihydroartemisinin (DHA)	Sigma-Aldrich®	Cat#1200520-200MG
Dimethyl sulfoxide (DMSO)	Sigma-Aldrich®	Cat#D2650
Endothelial cell growth supplement (ECGS)	ScienCell™ Research Laboratories	Cat#1052
Endothelial cell medium (ECM)	ScienCell™ Research Laboratories	Cat#1001
Fetal Bovine Serum (FBS) (ECM supplement)	ScienCell™ Research Laboratories	Cat#0025
Fetal Bovine Serum (FBS)	Gibco™	Cat#10500-064
Gelatin solution	Sigma-Aldrich®	Cat#G1393-100ml
Gentamicin	Sigma-Aldrich®	Cat#G1264-250MG
Glycerol	Sigma-Aldrich®	Cat#G7757-1L
Giemsa	Sigma-Aldrich®	Cat#48900-500ML-F
Hypoxanthine	Sigma-Aldrich®	Cat#H9377-25G
Immersion oil	Systerm	Cat#IM372-90
LD Columns	Miltenvi Biotec	Cat#130-042-091
L-Glutamine	Sigma-Aldrich®	Cat#G8540-100G
Methanol	Friendemann Schmidt	Cat#M2097-4-4001
Penicillin-Streptomycin Solution	Gibco	Cat#15140122
Penicillin-Streptomycin Solution (ECM supplement)	ScienCell™ Research Laboratories	Cat#0503
Percoll stock solution	Sigma-Aldrich®	Cat#P4937-500ML
RPMI 1640 medium	Gibco™	Cat#23400-013-1L
Sodium bicarbonate	Sigma-Aldrich®	Cat#S5761-500G
Sodium chloride	J.T.Baker	Cat#3624-69
Trypan blue	Sigma-Aldrich®	Cat#T6146
Trypsin solution	Sigma-Aldrich®	Cat# T3924-100ml
Ultrafiltration Spin Columns	biosharp®	Cat#BS-UFC-040-030
**Software**		
GraphPad Prism version 11.0.0	GraphPad	
**Others**		
0.5-10µL micropipette tips	Kirgen	Cat#KG5131-L
1.5mL microcentrifuge tube	GSBIO	Cat#CC102-N-F
1000µL micropipette filtered tips	Axygen	Cat#TF-1000-R-S
10mL serological pipette	Labselect	Cat#SP-013-10
15mL centrifuge tube	Biofil	Cat#CFT021500
200µL micropipette filtered tips	Axygen	Cat#TF-200-L-R-S
25mL serological pipette	Labselect	Cay#SP-013-25
25cm^2 cell culture flask canted neck (plug-seal cap)	SORFA	Cat#210110
3mL sterile transfer pipette	Biologix	Cat#30-0138A1
500mL vacuum bottle filter	Biofil	Cat#Fpe204500
50ml centrifuge tube	Biofil	Cat#CFT01115
50mL syringe	Terumo	Cat#SS*50LE
5mL serological pipette	Labselect	Cat#SP-013-5
75cm^2 cell culture flask canted neck (plug-seal cap)	SORFA	Cat#210200
96-well microplates	Nunclon Surface	Cat#137101
Cellulose acetate syringe filter (0.22um pore size)	Bioflow Lifescience	Cat#MALCA25022
Chamber slide	Lab-Tek II	Cat#154534PK
Carbon dioxide incubator	Lichen Scientific	Cat#LC-CBG-80S
Cryovial	Corning	Cat#430488
Falcon® Cell Culture Flask T25, filter cap	VWR™	Cat#29185298
Glass slide	Sail brand	Cat#7101
Hemocytometer	WATSON BIO LAB	Cat#177-112C
Kimwipes	Kim Tech Science Brand	Cat#34155/34120

LD columns	Miltenyi Biotec	Cat#130-042-901
Microscope slide with frosted side	Citoglas	Cat#P/N.0312-2101
Parafilm	Bemis	Cat#PM-996
QuadroMACS™ separator	Miltenyi Biotec	Cat#130-090-976
Vacutainer	BD	Cat#367284
